# Effect of Age and Age‐Defined Menopausal Status on Linaclotide Efficacy and Safety in Adults With Irritable Bowel Syndrome With Constipation or Chronic Idiopathic Constipation: Post Hoc Analyses of Phase 2b/3 Studies

**DOI:** 10.1111/nmo.70429

**Published:** 2026-09-06

**Authors:** Lin Chang, Kyle Staller, Lesley A. Houghton, Niha Yerneni, Sally Higgins, Wendy Chen, James Wu, Alexander C. Ford, Paul Feuerstadt, Darren M. Brenner

**Affiliations:** ^1^ Vatche and Tamar Manoukian Division of Digestive Diseases David Geffen School of Medicine, University of California Los Angeles California USA; ^2^ Division of Gastroenterology Massachusetts General Hospital Boston Massachusetts USA; ^3^ Division of Gastroenterology, Hepatology and Healthcare Acquired Infection, Leeds Institute of Medical Research University of Leeds Leeds UK; ^4^ Ironwood Pharmaceuticals, Inc. Boston Massachusetts USA; ^5^ AbbVie Inc. Chicago Illinois USA; ^6^ Yale School of Medicine New Haven Connecticut USA; ^7^ Physicians Alliance of Connecticut (PACT) Gastroenterology Center Hamden Connecticut USA; ^8^ Northwestern University Feinberg School of Medicine Chicago Illinois USA

**Keywords:** age, chronic idiopathic constipation, elderly, irritable bowel syndrome with constipation, linaclotide, menopause

## Abstract

**Background:**

This post hoc study explored the effect of age and age‐defined menopausal status on linaclotide efficacy and safety in adults with irritable bowel syndrome with constipation (IBS‐C) or chronic idiopathic constipation (CIC).

**Methods:**

Data were pooled from eight randomized, placebo‐controlled studies of linaclotide 72 μg (CIC), 145 μg (CIC), or 290 μg (IBS‐C). Patients were stratified by age (< 65 years; ≥ 65 years) and female patients by age‐defined menopausal status (premenopausal: ≤ 45 years; postmenopausal: > 45 years) as a surrogate for clinically confirmed menopausal status. Efficacy endpoints included change from baseline and time to response in complete spontaneous bowel movement (CSBM) frequency and abdominal symptoms.

**Results:**

Linaclotide increased weekly CSBM frequency from baseline versus placebo across age (IBS‐C, < 65 years, placebo: 0.8; 290 μg: 2.3; ≥ 65 years, placebo: 0.9; 290 μg: 2.6; CIC, < 65 years, placebo: 0.7; 72 μg: 1.7; 145 μg: 2.0; ≥ 65 years, placebo: 0.9; 72 μg: 1.5; 145 μg: 2.3) and menopausal subgroups. Abdominal symptoms reduced from baseline versus placebo across all subgroups. Median time (weeks) to an increase from baseline in weekly CSBM rate of ≥ 1 was shorter with linaclotide versus placebo across age (IBS‐C, < 65 years, placebo: 4.0; 290 μg: 2.0; ≥ 65 years, placebo: 3.0; 290 μg: 2.0; CIC, < 65 years, placebo: 4.0; 72 μg: 3.0; 145 μg: 2.0; ≥ 65 years, placebo: 5.0; 72 μg: 3.0; 145 μg: 1.5) and age‐defined menopausal subgroups.

**Conclusion:**

In this exploratory post hoc analysis, linaclotide was associated with improvements in bowel and abdominal symptoms across age and age‐defined menopausal subgroups, with a safety profile consistent with previous studies, suggesting that age and menopausal status did not meaningfully influence treatment outcomes.

## Key Points

- Pooled data from eight randomized, placebo‐controlled studies demonstrated that taking linaclotide improved various symptoms of irritable bowel syndrome with constipation (IBS‐C) or of chronic idiopathic constipation (CIC) compared with taking placebo, regardless of a patient's age or whether they were age‐defined to be premenopausal or postmenopausal.
- Patients with IBS‐C typically had improvement in bowel movements within about 2 weeks of starting linaclotide treatment regardless of age or age‐defined menopausal status. Patients with CIC had a dose‐dependent improvement in bowel movements, which happened earlier in those taking the higher linaclotide dose (145 μg) after about 1.5–2 weeks of treatment.
- Linaclotide was generally well tolerated across age and age‐defined menopausal subgroups, with a safety profile consistent with the known safety profile of linaclotide. Diarrhea was the most common adverse event.

## Plain Language Summary

Data from eight randomized, placebo‐controlled studies showed that linaclotide improved symptoms of irritable bowel syndrome with constipation (IBS‐C) and chronic idiopathic constipation (CIC) compared with placebo, across age and age‐defined menopausal subgroups. Patients with IBS‐C typically experienced improvements in bowel movements within 2 weeks of starting treatment, and in those with CIC, bowel movement improvements occurred within 1.5–3.0 weeks following initiation of linaclotide treatment, with earlier responses seen at higher doses. Improvements in abdominal pain, discomfort, and bloating were also observed. The safety profile of linaclotide was generally consistent across all patient groups, and linaclotide was well‐tolerated. Diarrhea was the most common adverse event and the leading cause of treatment discontinuation. Overall, these findings support linaclotide as an effective treatment option for adults with IBS‐C or CIC across age and age‐defined menopausal subgroups.

## Graphical abstract

A post hoc pooled analysis of eight phase 2b/3 trials showed that linaclotide improved bowel and abdominal symptoms and was associated with a shorter time to response than placebo in adults with IBS‐C or CIC, irrespective of age or age‐defined menopausal status.

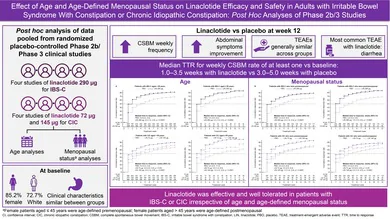

## 1 Introduction

Human physiological functions change with age and are further affected in women by menopause [1, 2]. Such changes may alter the body's response to medications, and affect drug absorption, distribution, metabolism, and elimination [3]. In general, the prevalence of irritable bowel syndrome (IBS) decreases with age, although this may be related to changes in pain perception, with older patients experiencing reduced levels of stress and reduced visceral hypersensitivity [4, 5, 6]. Indeed, younger patients with IBS with constipation (IBS‐C) report more severe gastrointestinal symptoms (i.e., abdominal distention and pain), whereas older patients report more frequent constipation [4, 7]. In an analysis of 2292 patients from studies in IBS‐C (87% of whom were female), peak abdominal symptom severity occurred in those aged ≥ 50 to < 60 years [8]. The prevalence and impact of some disorders of gut–brain interaction are higher in individuals aged < 65 years [9, 10].

However, the prevalence of chronic idiopathic constipation (CIC) increases with age, particularly in those older than 65 years, with reduced physical activity and polypharmacy often identified as contributing factors [11, 12]. Straining is the predominant symptom for elderly patients with CIC, occurring in up to 65% of individuals aged > 65 years [12, 13].

Depending on diagnostic criteria and population studied, the prevalence of IBS and CIC in adults in the USA based on Rome II–IV diagnostic criteria approximates to 1.3%–2.3% and 4.9%–19.4%, respectively [14, 15]. Furthermore, both IBS‐C and CIC are more common in women than in men. In prevalence studies, women with IBS were more than two times as likely as men to have IBS‐C and were also more than two times as likely as men to have CIC [16, 17]. However, data pertaining to the effect of menopause on IBS‐C and CIC are limited and contradictory [18, 19, 20, 21].

Linaclotide is a guanylate cyclase‐C agonist approved for the treatment of IBS‐C in adults (290 μg orally once daily) and CIC (72 μg or 145 μg orally once daily) [22]. With a well‐characterized safety profile, linaclotide has been shown to significantly improve abdominal and bowel symptoms for at least 12 weeks and up to 26 weeks in patients with IBS‐C [23, 24]; it has also been shown to significantly improve stool frequency and other bowel symptoms in patients with CIC [25, 26]. However, there is a lack of published data showing whether the efficacy and the safety profile of linaclotide change with aging or following menopause. This is of particular relevance given the increased incidence of CIC with advancing age and the higher prevalence of IBS‐C and CIC in women than in men [11, 16, 17]. In this post hoc analysis of data pooled from eight phase 2b/phase 3 clinical studies, we explored the efficacy and safety of linaclotide in adults with IBS‐C or CIC, stratified by age group (< 65 years and ≥ 65 years) and in women with IBS‐C or CIC (not taking contraception or hormone replacement therapy during the study intervention period) stratified by age‐defined menopausal status (age‐defined premenopausal: ≤ 45 years; age‐defined postmenopausal: > 45 years). Menopausal status was approximated using age as a surrogate measure, considering previously reported cutoffs and WHO epidemiological data [27, 28]. We hypothesized that linaclotide treatment effects would be observed across age and age‐defined menopausal status without evidence of clinically meaningful differences in response.

## 2 Materials and Methods

### 2.1 Study Design and Patients

In this post hoc analysis, data were pooled from eight randomized, double‐blind, placebo‐controlled trials of adults with IBS‐C or CIC: four IBS‐C trials (one phase 2b [NCT02559206] trial [29] and three phase 3 [NCT00948818; NCT00938717; NCT03573908] trials [23, 24, 30]); and four phase 3 CIC trials (NCT00730015; NCT00765882; NCT01642914; NCT02291679) [25, 26, 31]. The contributing studies were selected because of their broadly comparable study designs, eligibility criteria, treatment durations, and efficacy endpoints, thereby supporting pooled descriptive analyses. Study designs, inclusion and exclusion criteria, and results of these trials have been reported previously [23, 24, 25, 26, 29, 30, 31]. In the IBS‐C trials, patients received either a placebo or linaclotide 290 μg once daily. In the CIC studies, patients received either a placebo or linaclotide 72 μg or 145 μg once daily. In all trials, patients received treatment for at least 12 weeks, and data from these 12‐week treatment periods are reported herein.

Eligible patients were adults who met Rome II/III criteria for IBS‐C or CIC. Patients in the IBS‐C trials also had to have a baseline mean abdominal pain score of ≥ 3 (0–10 scale) at its worst. Additionally, patients were included if they reported less than three spontaneous bowel movements (SBMs) per week and at least one additional bowel symptom (straining, lumpy or hard stools, and/or sensation of incomplete evacuation) during > 25% of bowel movements for at least 12 weeks. Patients also needed to report SBMs and complete SBMs (CSBMs) below a certain threshold per week during the 2‐week baseline period. Patients in the IBS‐C trials needed to report no more than five SBMs and less than three CSBMs per week (except those in NCT02559206 and NCT03573908, who needed to report no more than 10 SBMs and no more than six CSBMs in the 14‐day baseline period); CIC trials required no more than six SBMs and less than three CSBMs per week. Patients with a history of loose or watery stools (Bristol Stool Form Scale score of 6 or 7) for > 25% of bowel movements during the 12 weeks before the screening visit were not eligible for inclusion. Prior medication use was recorded at the screening visit, and concomitant medication was evaluated at each visit. Protocol‐permitted laxatives (bisacodyl 5 mg tablets or bisacodyl 10 mg suppositories) were permitted as rescue medicine during the pretreatment and treatment periods if ≥ 72 h had passed since the previous bowel movement or if symptoms became intolerable. To be eligible for randomization, rescue medication should not have been used on the calendar day before or on the day of the randomization visit.

### 2.2 Assessments

All patients provided daily reports of their abdominal symptoms (pain, discomfort, and bloating) at their worst and weekly CSBM frequency rates. Abdominal pain, discomfort, and bloating were evaluated using either a 5‐point ordinal scale (1 = none; 5 = very severe) or an 11‐point numerical rating scale (0 = none; 10 = very severe), depending on the trial; before pooling, results reported using the 11‐point scale were converted to results on the 5‐point scale (i.e., 0 = “1” [none]; 1–3 = “2” [mild]; 4–6 = “3” [moderate]; 7–8 = “4” [severe]; and 9–11 = “5” [very severe]). Daily overall abdominal symptom score was calculated as the average of the daily abdominal pain, bloating, and discomfort scores. In all the IBS‐C and CIC trials, stool consistency was measured daily using the 7‐point ordinal Bristol Stool Form Scale (1 = separate hard lumps like nuts [difficult to pass]; 7 = watery, no solid pieces [entirely liquid]). Constipation and straining severity were measured weekly and daily, respectively, using a 5‐point ordinal scale, with higher scores indicating greater severity. In the IBS‐C trials, abdominal pain and constipation (APC + 1) responders were defined as those who, for at least 6 of the 12 weeks of treatment, had an increase from baseline of at least one CSBM and a decrease of ≥ 30% in their abdominal pain score during the same week. Safety assessments included the frequency of treatment‐emergent adverse events (TEAEs) and serious adverse events.

### 2.3 Statistical Analysis

Analyses of pooled data were conducted separately for IBS‐C and CIC. Analyses were performed in patients aged < 65 years and ≥ 65 years and among eligible females, according to age‐defined pre‐ or post‐menopausal status (≤ 45 years and > 45 years, respectively) who did not receive contraception or hormone replacement therapy during the study intervention period. The cutoff age of 45 years was identified through an age distribution of childbearing potential (defined as being not postmenopausal or not having undergone bilateral oophorectomy, hysterectomy, or tubal ligation) across the age range 40–60 years (Figure S1) and considering previously reported cutoffs and WHO criteria [27, 28]. This approach was intended to facilitate exploratory subgroup analyses rather than to establish clinically confirmed menopausal status. These subgroup analyses were exploratory in nature and were intended to evaluate whether treatment effects appeared to differ across age‐defined subgroups. No adjustments for multiplicity were performed, and formal treatment‐by‐subgroup interaction testing was not conducted.

All efficacy endpoints were assessed in the modified intent‐to‐treat population, which included all randomized patients who received at least one dose of study treatment and had available data at baseline and either during the 12‐week treatment period or at week 12, depending on the analysis methods used. Any patients who did not have post‐baseline data were excluded from the analyses. All safety assessments were performed in the safety population, which included all randomized patients who received at least one dose of study treatment.

Time to response was analyzed using the Kaplan–Meier method, and the corresponding descriptive *p* values were generated based on the log‐rank test. Weekly abdominal symptom scores and CSBM frequency were calculated as the means of the non‐missing daily scores and the weekly CSBM rates, respectively. Least squares (LS) mean change from baseline in abdominal symptoms, abdominal scores, and CSBM frequency over the 12‐week treatment period or at week 12 were evaluated. For the CIC trials, an analysis of covariance (ANCOVA) model was used and included fixed effects for the treatment group and geographic region, with the corresponding baseline value as a covariate [25, 26, 31]. For the IBS‐C trials, a mixed model for repeated measures (MMRM) framework was used with week (categorical), treatment, and week‐by‐treatment as fixed effects, patient as a random effect, and baseline value as a covariate [23, 24, 29, 30]. The corresponding two‐sided descriptive *p* values were calculated. The proportion of responders was compared between the treatment and placebo groups. The Cochran–Mantel–Haenszel test was stratified by geographic region. All *p* values were descriptive.

## 3 Results

### 3.1 Demographics and Baseline Characteristics

The pooled analysis of the IBS‐C trials comprised 2185 patients aged < 65 years and 163 patients aged ≥ 65 years; the pooled analysis of the CIC trials comprised 2148 patients aged < 65 years and 252 aged ≥ 65 years (Figure S2). Of the 1608 eligible women in the IBS‐C studies, 801 were age‐defined premenopausal (aged ≤ 45 years) and 807 age‐defined postmenopausal (aged > 45 years); of the 1654 eligible women in the CIC studies, 700 were classified as age‐defined premenopausal and 954 as age‐defined postmenopausal. Patient demographics and baseline clinical characteristics are presented in Table S1. Baseline demographic and disease characteristics were generally balanced across the pooled studies, supporting interpretation of the combined analyses. Overall, most patients were female (85.2%) and white (72.7%). Mean ages in years (standard deviation) of those age‐defined as postmenopausal were 56.8 (7.75), 56.6 (7.68), and 55.6 (7.31) in the linaclotide 72 μg, 145 μg, and 290 μg once daily groups, respectively. Baseline clinical characteristics, including severity of abdominal symptoms and CSBM frequency, were numerically comparable between age groups and menopausal status groups. Most patients with IBS‐C or CIC received concomitant medications. The proportion of patients receiving concomitant medications was higher in the ≥ 65 years group than in the < 65 years group (IBS‐C: 96.2% vs. 84.3% and CIC: linaclotide 72 μg group, 91.7% vs. 61.9%; linaclotide 145 μg group, 96.2% vs. 74.8%). This proportion was also higher in patients considered age‐defined postmenopausal than in those considered age‐defined premenopausal (IBS‐C: 89.3% vs. 79.6% and CIC: linaclotide 72 μg group, 74.1% vs. 50.8%; linaclotide 145 μg group, 84.0% vs. 68.0%).

### 3.2 Efficacy of Linaclotide by Age Group

**FIGURE 1:**
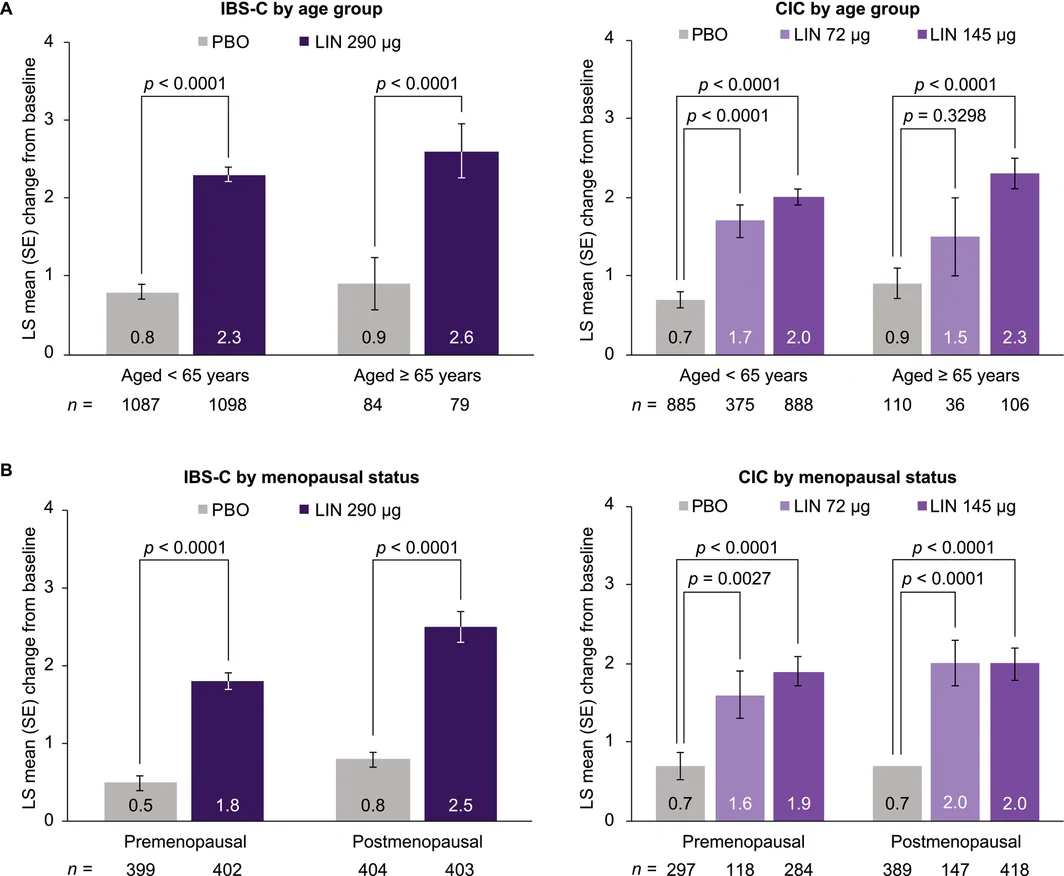
LS mean (SE) change from baseline at week 12 in weekly CSBM rate in patients with IBS‐C and in patients with CIC by (A) age group and (B) age‐defined menopausal status^†^. *p* values were based on an analysis of covariance (ANCOVA) model for the CIC trials and a mixed model for repeated measures (MMRM) framework for the IBS‐C trials. Data are for the mITT population, defined as all randomized patients who received at least one dose of study treatment and who had post‐baseline efficacy measurements. ^†^Female patients aged ≤ 45 years were age‐defined premenopausal; female patients aged > 45 years were age‐defined postmenopausal. CIC, chronic idiopathic constipation; CSBM, complete spontaneous bowel movement; IBS‐C, irritable bowel syndrome with constipation; LIN, linaclotide; LS, least‐squares; mITT, modified intent‐to‐treat; PBO, placebo; SE, standard error.

**FIGURE 2:**
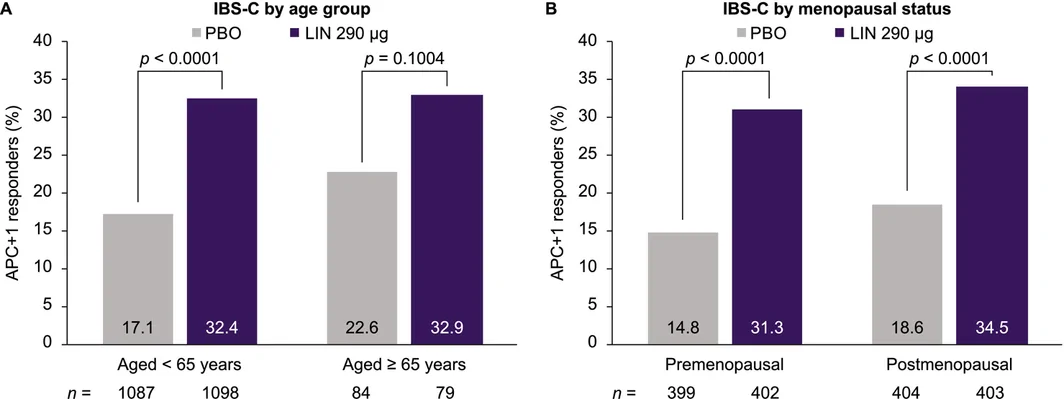
APC + 1 responder rates for patients with IBS‐C stratified by (A) age group and (B) age‐defined menopausal status^†^. A patient was considered an APC + 1 responder if, for at least 6 of the 12 weeks of the treatment, they experienced an increase of at least one CSBM from baseline and a decrease of ≥ 30% in their abdominal pain score during a particular week. *p* values are based on the Cochran–Mantel–Haenszel test controlling for geographic region. Data are for the mITT population, defined as all randomized patients who received at least one dose of study treatment and who had post‐baseline efficacy measurements. ^†^Female patients aged ≤ 45 years were age‐defined premenopausal; female patients aged > 45 years were age‐defined postmenopausal. APC, abdominal pain and constipation; CSBM, complete spontaneous bowel movement; IBS‐C, irritable bowel syndrome with constipation; LIN, linaclotide; modified intent‐to‐treat; PBO, placebo.

In patients with IBS‐C or CIC, linaclotide treatment increased CSBM frequency compared with placebo in both age groups (Figure 1A). The LS mean change from baseline (standard error) in CSBM frequency with linaclotide 290 μg was 2.3 (0.1) per week in patients with IBS‐C aged < 65 years and 2.6 (0.4) per week in those aged ≥ 65 years (*p* < 0.0001 vs. placebo for both age groups). Improvements were also observed in patients with CIC (Figure 1A). In patients with IBS‐C, the proportion of APC + 1 responders was statistically higher with linaclotide 290 μg than with placebo in patients aged < 65 years (356 [32.4%] vs. 186 [17.1%]; odds ratio 2.34; 95% confidence interval 1.91, 2.86; *p* < 0.0001) and was numerically higher in those aged ≥ 65 years (26 [32.9%] vs. 19 [22.6%]; odds ratio 1.80; 95% confidence interval 0.89, 3.62; *p* = 0.1004; Figure 2A). In patients with IBS‐C, linaclotide treatment was associated with greater reductions from baseline, compared with placebo, in abdominal pain, discomfort, and bloating scores and in the overall abdominal symptom score, irrespective of age group (Table 1). These reductions were also observed in patients with CIC (Table 1).

**TABLE 1:** Change from baseline at 12 weeks in abdominal symptoms by age group and by age‐defined menopausal status^a^ in patients with IBS‐C or CIC (MITT population).

	Patients with IBS‐C				Patients with CIC					
	Aged < 65 years (*n* = 2185)		Aged ≥ 65 years (*n* = 163)		Aged < 65 years (*n* = 2148)			Aged ≥ 65 years (*n* = 252)		
	PBO (*n* = 1087)	LIN 290 μg (*n* = 1098)	PBO (*n* = 84)	LIN 290 μg (*n* = 79)	PBO (*n* = 885)	LIN 72 μg (*n* = 375)	LIN 145 μg (*n* = 888)	PBO (*n* = 10)	LIN 72 μg (*n* = 36)	LIN 145 μg (*n* = 106)
Age group LS mean (SE)										
Abdominal pain score	−1.1 (0.1)	−1.9 (0.1)***	−1.2 (0.2)	−1.9 (0.2)*	−0.4 (0.0)	−0.5 (0.0)*	−0.5 (0.0)***	−0.4 (0.1)	−0.6 (0.1)	−0.5 (0.1)
Abdominal discomfort score	−1.2 (0.1)	−1.9 (0.1)***	−1.2 (0.2)	−1.9 (0.2)*	−0.4 (0.0)	−0.5 (0.0)*	−0.6 (0.0)***	−0.4 (0.1)	−0.6 (0.1)	−0.5 (0.1)
Abdominal bloating score	−1.1 (0.1)	−1.9 (0.1)***	−1.0 (0.2)	−1.9 (0.2)**	−0.4 (0.0)	−0.5 (0.0)*	−0.6 (0.0)***	−0.3 (0.1)	−0.4 (0.1)	−0.5 (0.1)*
Overall abdominal symptom score	−1.2 (0.1)	−1.9 (0.1)***	−1.1 (0.2)	−1.9 (0.2)*	−0.4 (0.0)	−0.5 (0.0)*	−0.6 (0.0)***	−0.5 (0.1)	−0.7 (0.1)	−0.6 (0.1)*

	Age‐defined premenopausal (*n* = 801)		Age‐defined postmenopausal (*n* = 807)		Age‐defined premenopausal (*n* = 699)^b^			Age‐defined postmenopausal (*n* = 954)		
	PBO (*n* = 399)	LIN 290 μg (*n* = 402)	PBO (*n* = 404)	LIN 290 μg (*n* = 403)	PBO (*n* = 297)	LIN 72 μg (*n* = 118)	LIN 145 μg (*n* = 284)	PBO (*n* = 389)	LIN 72 μg (*n* = 147)	LIN 145 μg (*n* = 418)
Age‐defined menopausal status group LS mean (SE)										
Abdominal pain score	−0.7 (0.1)	−1.6 (0.1)***	−1.1 (0.1)	−1.8 (0.1)***	−0.4 (0.0)	−0.6 (0.1)*	−0.6 (0.0)**	−0.4 (0.0)	−0.5 (0.1)	−0.5 (0.0)**
Abdominal discomfort score	−0.8 (0.1)	−1.7 (0.1)***	−1.2 (0.1)	−1.9 (0.1)***	−0.4 (0.0)	−0.6 (0.1)*	−0.6 (0.0)***	−0.4 (0.0)	−0.5 (0.1)	−0.6 (0.0)**
Abdominal bloating score	−0.8 (0.1)	−1.7 (0.1)***	−1.1 (0.1)	−1.9 (0.1)***	−0.4 (0.0)	−0.6 (0.1)*	−0.6 (0.1)***	−0.4 (0.1)	−0.5 (0.1)*	−0.6 (0.0)***
Overall abdominal symptom score	−0.8 (0.1)	−1.7 (0.1)***	−1.1 (0.1)	−1.9 (0.1)***	−0.4 (0.0)	−0.6 (0.1)*	−0.6 (0.0)***	−0.4 (0.0)	−0.5 (0.1)	−0.6 (0.0)***

*Note:* *p < 0.05; **p < 0.001; ***p < 0.0001 versus placebo; Descriptive *p* values for the difference in LS mean change from baseline in IBS‐C or CIC symptoms for linaclotide versus placebo were calculated. Data are for the mITT population, defined as all randomized patients who received at least one dose of study treatment and who had post‐baseline efficacy measurements. For each outcome, values are presented for patients with available change from baseline data for that outcome. An MMRM framework was used for IBS‐C (fixed effects: treatment, week, week‐by‐treatment interaction; random effect: patient; covariate: baseline). For CIC, an ANCOVA model included treatment, baseline stratum, region, and baseline value as a covariate.

Abbreviations: ANCOVA, analysis of covariate; CIC, chronic idiopathic constipation; IBS‐C, irritable bowel syndrome with constipation; LIN, linaclotide; LS, least‐squares; mITT, modified intent‐to‐treat; MMRM, mixed model for repeated measures; PBO, placebo; SE, standard error.

^a^ Female patients aged ≤ 45 years were age‐defined premenopausal; female patients aged > 45 years were age‐defined postmenopausal.

^b^ Data were unavailable for one patient with CIC who was considered premenopausal and randomized to the placebo arm.

**FIGURE 3:**
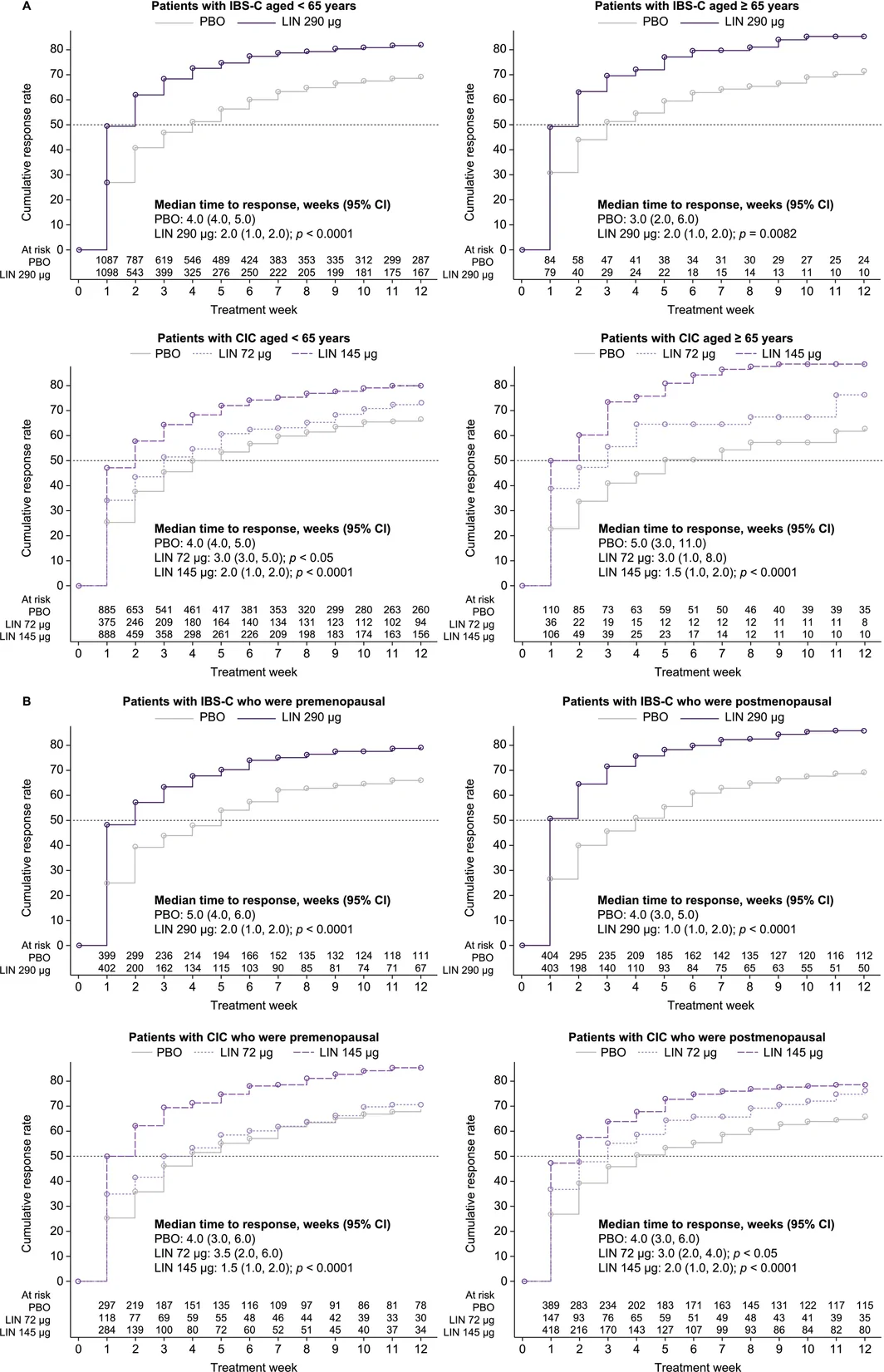
Time to improvement in CSBM weekly rate of at least one compared with baseline in patients with IBS‐C and in patients with CIC by (A) age group and (B) age‐defined menopausal status^†^. *n* is the number of patients at risk. Data are for the mITT population, defined as all randomized patients who received at least one dose of study treatment and who had post‐baseline efficacy measurements. An event was defined as having occurred on the first week that a patient had an increase from baseline in CSBM weekly rate of at least one. Open circles indicate censored patients. *p* values are based on log‐rank tests. ^†^Female patients aged ≤ 45 years were age‐defined premenopausal; female patients aged > 45 years were age‐defined postmenopausal. CI, confidence interval; CIC, chronic idiopathic constipation; CSBM, complete spontaneous bowel movement; IBS‐C, irritable bowel syndrome with constipation; mITT, modified intent‐to‐treat; LIN, linaclotide; PBO, placebo.

Patients treated with linaclotide experienced a shorter median response time for improvement from baseline in terms of frequency of CSBM increasing by at least one per week compared with patients receiving placebo, irrespective of age group (Figure 3A; Table 2). In patients with IBS‐C, the median time to improvement (95% confidence interval) in weekly CSBM rate was 2 (1, 2) weeks with linaclotide, irrespective of age group, compared with that with placebo of 4 (4, 5) weeks in patients aged < 65 years (*p* < 0.0001) and 3 (2, 6) weeks in those aged ≥ 65 years (*p* = 0.0082). Numerically similar improvements were observed in both age groups for patients with CIC, with the shortest median time to improvement reported for patients treated with the higher linaclotide dose (145 μg). For those aged < 65 years, the median time to improvement (95% confidence interval) was 2 (1, 2) weeks with linaclotide 145 μg and 4 (4, 5) weeks with placebo; for those aged ≥ 65 years, the median time to improvement was 1.5 (1, 2) weeks with linaclotide 145 μg and 5 (3, 11) weeks with placebo.

**TABLE 2:** Time to improvement from baseline in abdominal symptoms^a^ by age group and by age‐defined menopausal status^b^ in patients with IBS‐C or with CIC (mITT population).

Median time to response, weeks (95% CI)	Patients with IBS‐C				Patients with CIC					
	Aged < 65 years		Aged ≥ 65 years		Aged < 65 years			Aged ≥ 65 years		
	PBO	LIN 290 μg	PBO	LIN 290 μg	PBO	LIN 72 μg	LIN 145 μg	PBO	LIN 72 μg	LIN 145 μg
Weekly CSBM + ≥ 1 frequency^c^	4.0 (4.0, 5.0) *n* = 1087	2.0 (1.0, 2.0)*** *n* = 1098	3.0 (2.0, 6.0) *n* = 84	2.0 (1.0, 2.0)* *n* = 79	4.0 (4.0, 5.0) *n* = 885	3.0 (3.0, 5.0)* *n* = 375	2.0 (1.0, 2.0)*** *n* = 888	5.0 (3.0, 11.0) *n* = 110	3.0 (1.0, 8.0) *n* = 36	1.5 (1.0, 2.0)*** *n* = 106
Abdominal symptoms	8.0 (7.0, 9.0) *n* = 1080	4.0 (3.0, 4.0)*** *n* = 1091	8.0 (5.0, −) *n* = 84	3.0 (2.0, 5.0)* *n* = 78	– (−, −) *n* = 884	– (12.0, −) *n* = 375	9.0 (8.0, 12.0)*** *n* = 885	– (−, −) *n* = 110	– (7.0, −) *n* = 36	8.0 (5.0, −)* *n* = 106
Abdominal pain	6.0 (5.0, 7.0) *n* = 1085	3.0 (−, −)*** *n* = 1098	5.5 (4.0, −) *n* = 84	3.0 (2.0, 5.0)* *n* = 79	– (−, −) *n* = 765	12.0 (8.0, −) *n* = 290	9.0 (7.0, 10.0)*** *n* = 762	12.0 (7.0, −) *n* = 84	– (3.0, −) *n* = 22	6.0 (3.0, −) *n* = 73
Abdominal discomfort	7.0 (6.0, 8.0) *n* = 1078	3.0 (3.0, 4.0)*** *n* = 1089	8.0 (4.0, −) *n* = 84	3.0 (2.0, 4.0)* *n* = 78	– (11.0, −) *n* = 765	11.0 (8.0, −) *n* = 290	7.0 (5.0, 9.0)*** *n* = 762	– (7.0, −) *n* = 84	5.0 (2.0, −) *n* = 22	7.0 (3.0, −) *n* = 73
Abdominal bloating	8.0 (7.0, 10.0) *n* = 1067	4.0 (3.0, 5.0)*** *n* = 1082	11.0 (6.0, −) *n* = 83	3.0 (2.0, 5.0)** *n* = 75	– (−, −) *n* = 765	12.0 (9.0, −)* *n* = 290	8.0 (6.0, 9.0)*** *n* = 762	– (−, −) *n* = 84	– (3.0, −) *n* = 22	9.0 (4.0, −)** *n* = 73

Median time to response, weeks (95% CI)	Age‐defined premenopausal		Age‐defined postmenopausal		Age‐defined premenopausal			Age‐defined postmenopausal		
	PBO	LIN 290 μg	PBO	LIN 290 μg	PBO	LIN 72 μg	LIN 145 μg	PBO	LIN 72 μg	LIN 145 μg
Weekly CSBM + ≥ 1 frequency^c^	5.0 (4.0, 6.0) *n* = 399	2.0 (1.0, 2.0)*** *n* = 402	4.0 (3.0, 5.0) *n* = 404	1.0 (1.0, 2.0)*** *n* = 403	4.0 (3.0, 6.0) *n* = 297	3.5 (2.0, 6.0) *n* = 118	1.5 (1.0, 2.0)*** *n* = 284	4.0 (3.0, 6.0) *n* = 389	3.0 (2.0, 4.0)* *n* = 147	2.0 (1.0, 2.0)*** *n* = 418
Abdominal symptoms	9.0 (8.0, 11.0) *n* = 397	4.0 (3.0, 4.0)*** *n* = 399	9.0 (6.0, 12.0) *n* = 403	4.0 (3.0, 4.0)*** *n* = 402	– (10.0, −) *n* = 296	12.0 (7.0, −) *n* = 118	8.0 (5.0, 10.0)* *n* = 283	– (−, −) *n* = 389	– (7.0, −)* *n* = 147	11.0 (8.0, −)*** *n* = 416
Abdominal pain	8.0 (6.0, 9.0) *n* = 392	3.0 (−, −)*** *n* = 400	6.0 (5.0, 9.0) *n* = 403	3.0 (3.0, 4.0)*** *n* = 403	– (8.0, −) *n* = 296	9.0 (7.0, −) *n* = 118	8.0 (5.0, 10.0)* *n* = 283	– (−, −) *n* = 389	11.0 (7.0, −) *n* = 147	9.0 (7.0, −)* *n* = 416
Abdominal discomfort	8.0 (7.0, 10.0) *n* = 395	3.0 (3.0, 4.0)*** *n* = 399	7.0 (5.0, 10.0) *n* = 403	3.0 (2.0, 4.0)*** *n* = 400	10.0 (7.0, −) *n* = 296	9.0 (6.0, −) *n* = 118	6.0 (5.0, 9.0)* *n* = 283	– (11.0, −) *n* = 389	10.0 (6.0, −)* *n* = 147	7.0 (6.0, 10.0)** *n* = 416
Abdominal bloating	9.0 (8.0, 12.0) *n* = 393	4.0 (0.30, 5.0)*** *n* = 395	10.0 (7.0, −) *n* = 399	4.0 (3.0, 5.0)*** *n* = 398	– (9.0, −) *n* = 296	10.0 (7.0, −) *n* = 118	5.0 (4.0, 7.0)*** *n* = 283	– (−, −) *n* = 389	12.0 (7.0, –)** *n* = 147	10.0 (7.0, −)*** *n* = 416

*Note:* *p < 0.05; **p < 0.001; ***p < 0.0001 versus placebo; *p* values are based on log‐rank tests. *n* is the number of patients at risk. Data are for the mITT population, defined as included all randomized patients who received at least one dose of study treatment who had post‐baseline efficacy measurements. '−' indicates that the median time to response or corresponding CI bound was not estimable within the 12‐week treatment period.

Abbreviations: CI, confidence interval; CIC, chronic idiopathic constipation; CSBM, complete spontaneous bowel movement; IBS‐C, irritable bowel syndrome with constipation; LIN, linaclotide; mITT, modified intent‐to‐treat; PBO, placebo.

^a^ Abdominal symptom response was defined as a reduction from baseline in abdominal pain, discomfort, or bloating score of ≥ 30%.

^b^ Female patients aged ≤ 45 years were age‐defined premenopausal; female patients aged > 45 years were age‐defined postmenopausal.

^c^ An event was defined as having occurred on the first week that a patient had an increase from baseline in weekly CSBM rate of at least one.

**FIGURE 4:**
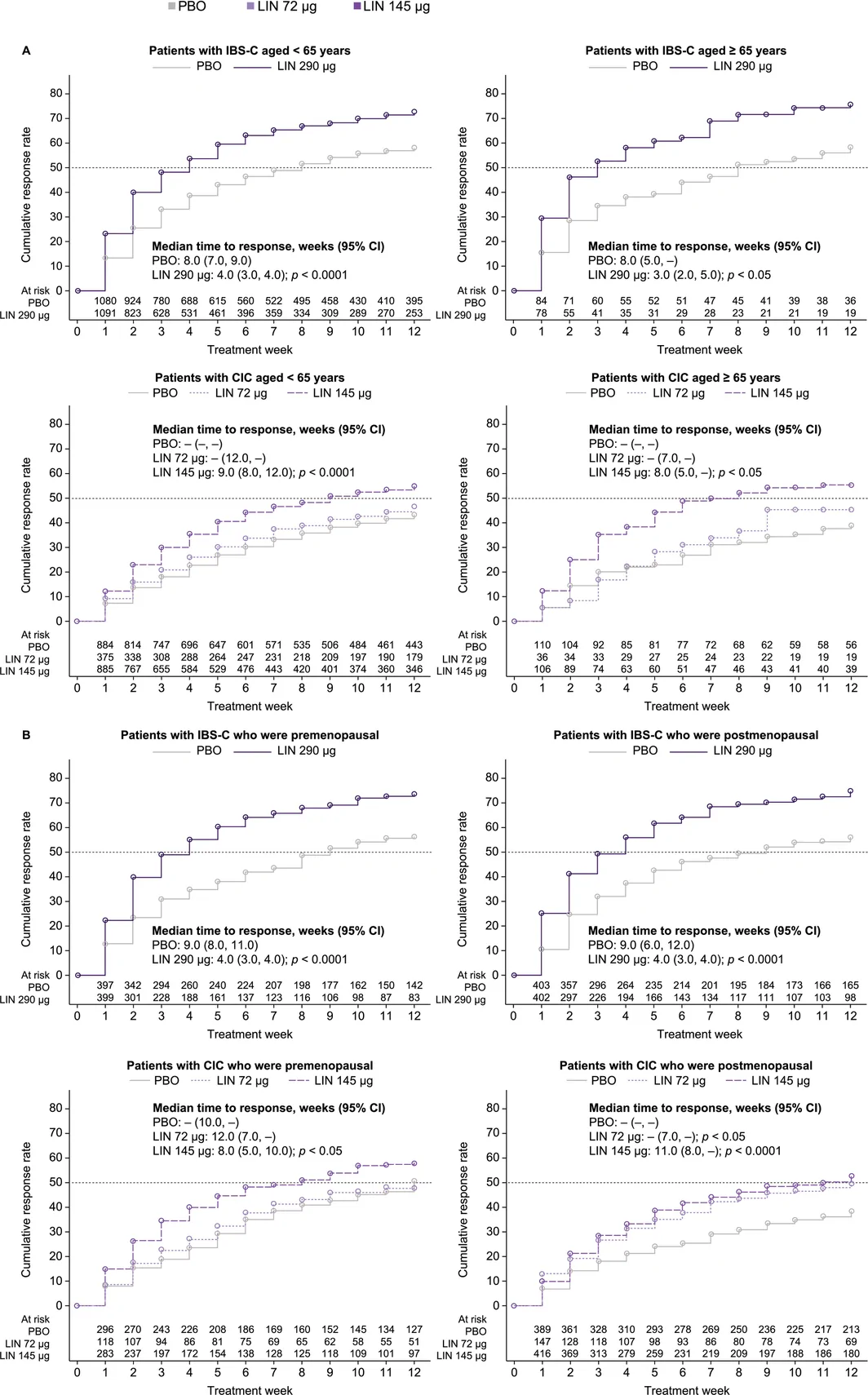
Time to improvement in abdominal symptom response in patients with IBS‐C and in patients with CIC by (A) age group and (B) age‐defined menopausal status^†^. *n* is the number of patients at risk. Data are for the mITT population, defined as all randomized patients who received at least one dose of study treatment and who had post‐baseline efficacy measurements. Abdominal symptom response was defined as a reduction from baseline in abdominal pain, abdominal discomfort, or abdominal bloating score of ≥ 30%. Open circles indicate censored patients. *p* values are based on log‐rank tests. ^†^Female patients aged ≤ 45 years were age‐defined premenopausal; female patients aged > 45 years were age‐defined postmenopausal. CI, confidence interval; CIC, chronic idiopathic constipation; IBS‐C, irritable bowel syndrome with constipation; mITT, modified intent‐to‐treat; LIN, linaclotide; PBO, placebo.

Similarly, the median time to response for improvements from baseline in abdominal score (Figure 4A) and in abdominal symptom scores (Table 2) was shorter in patients with IBS‐C treated with linaclotide than in those receiving placebo, regardless of age. In patients with CIC, measurable improvements in time to response were observed with linaclotide 145 μg for abdominal score, with favorable results evident for abdominal symptoms in both age groups (Figure 4A; Table 2).

### 3.3 Safety of Linaclotide by Age Group

A summary of TEAEs for patients with IBS‐C and CIC by age group is presented in Table 3. The proportions of patients treated with linaclotide who experienced at least one TEAE were comparable between the two age groups. Diarrhea was the most common TEAE in patients treated with linaclotide, irrespective of age. Diarrhea was experienced by 15.4% and 17.7% of patients with IBS‐C receiving linaclotide (290 μg) aged < 65 years and those aged ≥ 65 years, respectively. In patients with CIC receiving linaclotide 72 μg, 19.2% and 19.4% of patients in the < 65 years and ≥ 65 years groups, respectively, experienced diarrhea; in those receiving linaclotide 145 μg, 16.4% and 21.7% in the < 65 years and ≥ 65 years groups, respectively, experienced diarrhea. The proportion of patients with at least one TEAE leading to study drug discontinuation during the treatment period was low in both age groups. The highest rate of treatment discontinuation due to TEAEs occurred in the ≥ 65 years group of patients with IBS‐C receiving linaclotide (290 μg; 11.4%) (Table 3). Diarrhea led to the highest rate of discontinuation in each linaclotide group and was higher in those aged ≥ 65 years (0%–6.3%) than in those aged < 65 years (2.7%–4.1%).

**TABLE 3:** TEAEs in patients with IBS‐C or CIC by age group and by age‐defined menopausal status^a^ (safety population).

Patients, *n* (%)	Patients with IBS‐C (*N* = 2351)				Patients with CIC (*N* = 2402)					
	Aged < 65 years (*n* = 2188)		Aged ≥ 65 years (*n* = 163)		Aged < 65 years (*n* = 2150)			Aged ≥ 65 years (*n* = 252)		
	PBO (*n* = 1088)	LIN 290 μg (*n* = 1100)	PBO (*n* = 84)	LIN 290 μg (*n* = 79)	PBO (*n* = 887)	LIN 72 μg (*n* = 375)	LIN 145 μg (*n* = 888)	PBO (*n* = 110)	LIN 72 μg (*n* = 36)	LIN 145 μg (*n* = 106)
≥ 1 TEAE	495 (45.5)	566 (51.5)	37 (44.0)	46 (58.2)	340 (38.3)	131 (34.9)	424 (47.7)	52 (47.3)	12 (33.3)	56 (52.8)
≥ 1 TEAE leading to study discontinuation	17 (1.6)	75 (6.8)	4 (4.8)	9 (11.4)	24 (2.7)	12 (3.2)	49 (5.5)	6 (5.5)	0 (0)	8 (7.5)
TEAEs reported in ≥ 3% of patients in any LIN group, by preferred term										
Abdominal distension	12 (1.1)	21 (1.9)	0 (0)	1 (1.3)	13 (1.5)	9 (2.4)	19 (2.1)	0 (0)	0 (0)	4 (3.8)
Abdominal pain	30 (2.8)	45 (4.1)	1 (1.2)	0 (0)	19 (2.1)	6 (1.6)	25 (2.8)	3 (2.7)	0 (0)	3 (2.8)
Diarrhea	23 (2.1)	169 (15.4)	5 (6.0)	14 (17.7)	45 (5.1)	72 (19.2)	146 (16.4)	7 (6.4)	7 (19.4)	23 (21.7)
Flatulence	18 (1.7)	34 (3.1)	1 (1.2)	1 (1.3)	28 (3.2)	5 (1.3)	22 (2.5)	3 (2.7)	1 (2.8)	6 (5.7)
Headache	26 (2.4)	40 (3.6)	2 (2.4)	1 (1.3)	25 (2.8)	2 (0.5)	20 (2.3)	2 (1.8)	0 (0)	3 (2.8)
Nasopharyngitis	38 (3.5)	21 (1.9)	3 (3.6)	3 (3.8)	20 (2.3)	1 (0.3)	13 (1.5)	0 (0)	1 (2.8)	7 (6.6)
Nausea	47 (4.3)	44 (4.0)	2 (2.4)	2 (2.5)	21 (2.4)	1 (0.3)	25 (2.8)	3 (2.7)	0 (0)	1 (0.9)
Sinusitis	45 (4.1)	35 (3.2)	3 (3.6)	2 (2.5)	10 (1.1)	4 (1.1)	26 (2.9)	2 (1.8)	0 (0)	4 (3.8)
Upper respiratory tract infection	37 (3.4)	39 (3.5)	3 (3.6)	3 (3.8)	24 (2.7)	6 (1.6)	35 (3.9)	3 (2.7)	0 (0)	2 (1.9)
UTI	32 (2.9)	28 (2.5)	3 (3.6)	1 (1.3)	16 (1.8)	2 (0.5)	18 (2.0)	3 (2.7)	1 (2.8)	3 (2.8)

	Age‐defined premenopausal (*n* = 801)		Age‐defined postmenopausal (*n* = 808)		Age‐defined premenopausal (*n* = 701)			Age‐defined postmenopausal (*n* = 954)		
	PBO (*n* = 399)	LIN 290 μg (*n* = 402)	PBO (*n* = 405)	LIN 290 μg (*n* = 403)	PBO (*n* = 299)	LIN 72 μg (*n* = 118)	LIN 145 μg (*n* = 284)	PBO (*n* = 389)	LIN 72 μg (*n* = 147)	LIN 145 μg (*n* = 418)
≥ 1 TEAE	182 (45.6)	209 (52.0)	197 (48.6)	227 (56.3)	122 (40.8)	39 (33.1)	142 (50.0)	155 (39.8)	55 (37.4)	202 (48.3)
≥ 1 TEAE leading to study discontinuation	7 (1.8)	29 (7.2)	8 (2.0)	30 (7.4)	13 (4.3)	3 (2.5)	18 (6.3)	11 (2.8)	6 (4.1)	24 (5.7)
TEAEs reported in ≥ 3% of patients in any LIN group, by preferred term										
Abdominal distension	4 (1.0)	7 (1.7)	6 (1.5)	11 (2.7)	3 (1.0)	2 (1.7)	5 (1.8)	2 (0.5)	2 (1.4)	10 (2.4)
Abdominal pain	10 (2.5)	20 (5.0)	9 (2.2)	12 (3.0)	6 (2.0)	1 (0.8)	7 (2.5)	14 (3.6)	4 (2.7)	13 (3.1)
Diarrhea	6 (1.5)	54 (13.4)	10 (2.5)	69 (17.1)	13 (4.3)	20 (16.9)	42 (14.8)	21 (5.4)	33 (22.4)	78 (18.7)
Flatulence	6 (1.5)	14 (3.5)	8 (2.0)	13 (3.2)	8 (2.7)	4 (3.4)	6 (2.1)	10 (2.6)	2 (1.4)	17 (4.1)
Headache	10 (2.5)	16 (4.0)	8 (2.0)	14 (3.5)	13 (4.3)	0 (0)	9 (3.2)	11 (2.8)	2 (1.4)	10 (2.4)
Nasopharyngitis	15 (3.8)	7 (1.7)	13 (3.2)	10 (2.5)	11 (3.7)	0 (0)	4 (1.4)	4 (1.0)	0 (0)	11 (2.6)
Nausea	13 (3.3)	18 (4.5)	14 (3.5)	17 (4.2)	3 (1.0)	0 (0)	7 (2.5)	14 (3.6)	0 (0)	16 (3.8)
Sinusitis	17 (4.3)	15 (3.7)	16 (4.0)	10 (2.5)	4 (1.3)	1 (0.8)	13 (4.6)	4 (1.0)	2 (1.4)	9 (2.2)
Upper respiratory tract infection	14 (3.5)	15 (3.7)	17 (4.2)	22 (5.5)	9 (3.0)	3 (2.5)	15 (5.3)	13 (3.3)	1 (0.7)	12 (2.9)
UTI	13 (3.3)	20 (5.0)	10 (2.5)	7 (1.7)	7 (2.3)	1 (0.8)	4 (1.4)	7 (1.8)	2 (1.4)	15 (3.6)

*Note:* Safety population includes all randomized patients who received at least one dose of study treatment.

Abbreviations: CIC, chronic idiopathic constipation; IBS‐C, irritable bowel syndrome with constipation; LIN, linaclotide; PBO, placebo; TEAE, treatment‐emergent adverse effect; UTI, urinary tract infection.

^a^ Female patients aged ≤ 45 years were age‐defined premenopausal; female patients aged > 45 years were age‐defined postmenopausal.

### 3.4 Efficacy of Linaclotide by Age‐Defined Menopausal Status

Linaclotide treatment was associated with improvements in CSBM frequency compared with placebo in both menopausal status groups in patients with IBS‐C or CIC (Figure 1B). In patients with IBS‐C receiving linaclotide (290 μg), LS mean change from baseline (standard error) in CSBMs per week was 1.8 (0.1) and 2.5 (0.2) in patients considered premenopausal and those considered postmenopausal, respectively (*p* < 0.0001 vs. placebo for both status groups). In patients with CIC, LS mean changes from baseline (standard error) with linaclotide 72 μg and 145 μg were 1.6 (0.3) and 1.9 (0.2), respectively, in the age‐defined premenopausal group (*p* = 0.0027 and *p* < 0.0001 vs. placebo, respectively) and 2.0 (0.3) and 2.0 (0.2), respectively, in the age‐defined postmenopausal group (*p* < 0.0001 vs. placebo for both). In patients with IBS‐C, the proportion of APC + 1 responders was significantly greater (*p* < 0.0001) with linaclotide 290 μg than with placebo, regardless of age‐defined menopausal status (Figure 2B). Furthermore, in patients with IBS‐C and in those with CIC receiving the higher linaclotide dose (145 μg), compared with placebo, linaclotide was associated with greater reductions from baseline in abdominal pain, discomfort, and bloating scores, and in the overall abdominal symptom score, irrespective of age‐defined menopausal status (Table 1).

Similar to findings in the age group analyses, compared with placebo, linaclotide shortened the median time to response for an increase from baseline in weekly CSBM rate of at least one in both menopausal status groups in patients with IBS‐C or CIC (Figure 3B; Table 2). In patients with IBS‐C receiving linaclotide (290 μg), the median time (95% confidence interval) to an increase in the weekly CSBM rate of at least one was 2 (1, 2) weeks for age‐defined premenopausal patients and 1 (1, 2) week for age‐defined postmenopausal patients, compared with 5 (4, 6) weeks and 4 (3, 5) weeks, respectively, for those receiving placebo. In patients with CIC, the median response time for an increase in weekly CSBM rate of at least one was lower with linaclotide 72 μg or 145 μg than with placebo and was statistically significantly lower with linaclotide 145 μg (age‐defined premenopausal: 1.5 (1, 2) weeks vs. 4 (3, 6) weeks, *p* < 0.0001; age‐defined postmenopausal: 2 (1, 2) weeks vs. 4 (3, 6) weeks, *p* < 0.0001). In patients with IBS‐C and those with CIC receiving the higher linaclotide dose (145 μg), the median time to response for improvements from baseline in abdominal symptom score (Figure 4B) and abdominal symptoms (Table 2) was shorter with linaclotide than with placebo, regardless of age‐defined menopausal status.

### 3.5 Safety of Linaclotide by Age‐Defined Menopausal Status

The proportions of patients treated with linaclotide who experienced at least one TEAE were numerically comparable between menopausal groups in patients with IBS‐C or CIC (Table 3). In line with the analysis by age group, diarrhea was the most common TEAE with linaclotide treatment, irrespective of age‐defined menopausal status. For patients with IBS‐C treated with linaclotide (290 μg), diarrhea was reported in 13.4% of the age‐defined premenopausal group and in 17.1% of the age‐defined postmenopausal group. In patients with CIC receiving linaclotide 72 μg and those with CIC receiving linaclotide 145 μg, respectively, rates of diarrhea were 16.9% and 14.8% in the age‐defined premenopausal group and 22.4% and 18.7% in the age‐defined postmenopausal group (Table 3). The proportions of patients with at least one TEAE leading to study drug discontinuation during the treatment period were generally low and comparable for patients in both menopausal status groups. Diarrhea led to the highest rate of discontinuation in each linaclotide group and was higher in those considered postmenopausal (4.1%–4.5%) than in those considered premenopausal (1.7%–4.2%).

## 4 Discussion

This was a large, pooled analysis of eight phase 2b or phase 3 clinical trials comprising more than 4000 patients with IBS‐C or CIC in which the effect of linaclotide treatment was analyzed by age group and by age‐defined menopausal status. Given the well‐recognized effects of aging and menopause on gastrointestinal physiology and symptom presentation, understanding whether these factors may influence treatment response may help inform clinical‐decision making when selecting therapy for adults with IBS‐C or CIC. These pooled analyses demonstrated improvements in bowel and abdominal outcomes with linaclotide across age and age‐defined menopausal subgroups, and importantly, no qualitative evidence emerged to suggest that age or age‐defined menopausal status meaningfully altered treatment response. The results showed that in patients with IBS‐C or CIC, there was no obvious loss of efficacy observed across age groups (< 65 years or ≥ 65 years) and across age‐defined menopausal subgroups (≤ 45 years and > 45 years), with linaclotide increasing weekly CSBM frequency and shortening the time to improvement from baseline in weekly CSBM rate of at least one compared with placebo. These improvements with linaclotide treatment were accompanied by greater reductions from baseline, as well as a shortened time to response, in abdominal symptoms (pain, discomfort, and bloating) and the overall abdominal symptom score compared with placebo in the two age groups and in the age‐defined premenopausal and postmenopausal groups. Although disorders of gut–brain interaction are common at any age, studies have demonstrated that the prevalence of IBS‐C declines with advancing age, with younger adults reporting more severe gastrointestinal/abdominal symptoms [4, 7]. In contrast, the prevalence of constipation increases with age, particularly after the age of 65 years [11, 12]. Contributing factors to the more severe abdominal symptoms experienced by younger adults include increased visceral hypersensitivity, which is associated with abdominal pain intensity, and increased anxiety, which has been shown to be associated with abdominal bloating and pain in those with IBS‐C aged 20–49 years [4, 7]. Conversely, older adults may have less visceral hypersensitivity than younger adults, but lifestyle factors (e.g., reduced physical activity) and slower colonic transit may contribute to the increased severity of constipation experienced by older patients [4, 11]. The prevalence of both IBS‐C and CIC is reported to be higher in female individuals than in male individuals [16, 32], and severe constipation is more common in elderly women, with rates up to threefold higher than those seen in elderly men [33]. Hormonal changes related to menopause transition may influence gastrointestinal physiology and symptom perception. However, published data regarding the effect of hormonal changes on IBS‐C and CIC remain limited and are sometimes conflicting [8, 18, 27]. In a study by Lenhart et al. [27], postmenopausal women (those aged > 45 years and with an absence of menses for ≥ 1 year) reported more severe IBS symptoms overall compared with premenopausal women, although the authors found no difference between the two groups in terms of individual abdominal symptoms. A global epidemiology study that comprised 1390 women with IBS and 4049 women with CIC showed that premenopausal women with IBS or CIC experienced more frequent constipation‐related symptoms than postmenopausal women, although accidental stool leakage in IBS and digital manual maneuvers in CIC were higher in postmenopausal women than in premenopausal women [20]. These differences were generally not observed in similarly aged men (< 50 years vs. ≥ 50 years), suggesting that hormonal and/or pelvic floor factors, rather than age alone, may contribute to these symptom differences. An important consideration when interpreting these findings is that menopausal status was approximated using age rather than established using standard clinical criteria, such as at least 12 months of amenorrhea or hormonal assessment. Some women, especially those in the perimenopausal transition, may therefore have been misclassified. Although this limitation should be considered when interpreting subgroup findings, the analysis provides useful exploratory information regarding the potential influence of age‐related hormonal status on response to treatment with linaclotide.

Similar to the findings in this study on the efficacy of linaclotide by age group in CIC, other therapies, specifically, prucalopride, lubiprostone, elobixibat, and plecanatide, have been shown to be efficacious in increasing CSBMs and SBMs in adults aged ≥ 65 years [11]. Furthermore, in another previous study, an analysis of pooled data from four phase 3 trials in CIC and IBS‐C, it was reported that, compared with placebo, plecanatide significantly improved stool consistency and CSBM frequency from baseline at week 12 both in patients aged < 65 years and in those aged ≥ 65 years [34]. To our knowledge, this study is the first analysis evaluating the treatment response to linaclotide according to age‐defined menopausal status.

IBS‐C and CIC substantially impair patients' daily functioning, work productivity, and psychological well‐being, resulting in adverse effects on physical and mental health‐related quality of life [17, 35, 36]. Symptoms are burdensome and can affect work productivity, daily activities, and social relationships [36, 37]. In elderly patients, symptoms of constipation are associated with a deterioration in quality of life and a negative effect on mental health, whereas postmenopausal women with IBS have been shown to report significantly lower scores for physical health‐related quality of life than premenopausal women with IBS [11, 27]. Given this, it is important that patients receiving treatment for IBS‐C or CIC see timely improvements in their symptoms, especially because not doing so may affect treatment adherence [38, 39]. In this analysis, it was shown that, compared with placebo, linaclotide was associated with a shorter time to response for an increase from baseline in weekly CSBM rate of at least one and an improvement in abdominal score/symptoms in both age groups and both age‐defined menopausal status groups. These time‐to‐response analyses are crucial in discussions between clinicians and patients to help in setting appropriate treatment expectations around the timing of different symptom improvements, particularly given that abdominal symptom improvement can occur separately and after bowel symptom improvements [38, 39]. Managing treatment expectations and incorporating shared decision‐making are important for improving treatment adherence, which is vital for ensuring positive outcomes in chronic conditions such as IBS and CIC.

The safety findings in this analysis were consistent with the established safety profile of linaclotide in adults reported across previous phase 3 clinical trials and pooled analyses [40]. The safety profile of linaclotide was comparable across the two age groups and between the two age‐defined menopausal status groups, with diarrhea reported as the most frequent TEAE with linaclotide in all groups. Diarrhea was the most frequent cause of discontinuation in patients who received linaclotide; discontinuation due to diarrhea was slightly higher in patients aged ≥ 65 years than in those aged < 65 years and in patients age‐defined as postmenopausal than in those age‐defined as premenopausal. Although discontinuation rates due to diarrhea were slightly higher in older and age‐defined postmenopausal subgroups, the overall safety profile remained consistent with that reported in prior pooled analyses of linaclotide [40].

This study had several strengths and limitations. First, pooling patient‐level data from four trials in IBS‐C and four trials in CIC providing one of the largest datasets available to evaluate the age and age‐defined menopausal subgroup responses to linaclotide, with consistent endpoint definitions across studies, ensured a large patient population and a robust dataset for the analyses by age and by age‐defined menopausal status. In addition, to our knowledge, this is the first study to assess the efficacy of a treatment for IBS‐C and CIC according to patients' age‐defined menopausal status. The limitations of this study included its post hoc nature, the relatively small subgroup of patients with IBS‐C aged ≥ 65 years, and the potential for misclassification of menopausal status owing to the use of 45 years as a cutoff. We acknowledge that this cutoff is at the lower end of WHO epidemiological data for menopause (between 45 and 55 years for women worldwide) and that standard clinical criteria for menopause (e.g., at least 12 months of amenorrhea) was not collected, precluding assessment of associations with confirmed menopausal status and symptom severity. These factors could be a consideration for investigation in future studies. Additionally, the pooled study population was predominantly white and non‐Hispanic, with relatively limited representation of several racial and ethnic minority groups, particularly Asian. Therefore, although these findings are likely applicable to populations similar to those enrolled in the contributing trials, caution should be exercised when extrapolating these results to other race or ethnic populations. Future studies including more geographically and ethnically diverse populations would strengthen the generalizability of these findings. Finally, treatment effects were not formally assessed by individual study, and between‐study heterogeneity was not evaluated as part of this post hoc analysis. However, the pooled studies were intentionally selected because they shared comparable study designs, eligibility criteria, efficacy endpoints, treatment durations, and placebo‐controlled randomized designs, thereby supporting the use of pooled descriptive analyses despite the absence of formal heterogeneity testing. Although heterogeneity between studies cannot be excluded, we did not observe any qualitative evidence of inconsistent treatment effects across contributing studies.

## 5 Conclusions

In this large, pooled analysis of eight phase 2b or phase 3 trials, linaclotide was associated with clinically meaningful improvements in bowel and abdominal symptoms, with a shorter time‐to‐response across the age and age‐defined menopausal subgroups evaluated, compared with placebo. The safety profile remained consistent with previous studies, with no new safety findings identified. These exploratory findings suggest that age and age‐defined menopausal status are unlikely to meaningfully influence the efficacy or tolerability of linaclotide in adults with IBS‐C or CIC.

## Author Contributions

All authors had access to relevant data and participated in the drafting, review, and approval of this publication. No honoraria or payments were made for authorship.

## Funding

AbbVie and Ironwood Pharmaceuticals funded this study and participated in the study design, research, analysis, data collection, interpretation of data, reviewing, and approval of the publication.

## Conflicts of Interest

L.C. has served on scientific advisory boards for Alfasigma, Ardelyx, Atmo BioSciences, Ironwood Pharmaceuticals, and Vibrant Gastro; has served as a consultant for Bausch Health, Eli Lilly, FoodMarble Digestive Health, GlaxoSmithKline, and Ono Pharmaceuticals; as a speaker for Bausch Health, Medscape, and PICO Health; has received research support from AnX Robotica and Ironwood Pharmaceuticals; and has nonvested stock options with FoodMarble Digestive Health, ModifyHealth, PICO Health, and Trellus Health. K.S. has served as a consultant for AbbVie, Ardelyx, Atmo BioSciences, Ferring Pharmaceuticals, Gemelli Biotech, Laborie Medical Technologies, Salix Pharmaceuticals, and Takeda; serves on the medical advisory board of Mindset Health; and has received research support from Ardelyx. L.A.H. has served on a scientific advisory board for Ironwood Pharmaceuticals; has served on a scientific advisory board for GSK and Symprove, UK; and has acted as a consultant for Pfizer, USA. N.Y. was an employee of Ironwood Pharmaceuticals at the time of the study and may hold Ironwood Pharmaceuticals stock. S.H. and J.W. are full‐time employees of Ironwood Pharmaceuticals and may hold Ironwood Pharmaceuticals stock. W.C. is a full‐time employee of AbbVie and may hold AbbVie stock. A.C.F. has served on an advisory board for Ironwood Pharmaceuticals Inc. P.F. is on speaker bureaus for Ferring Pharmaceuticals, Nestle Health Sciences, Regeneron, and Sanofi, and is on advisory boards for Acurx Pharmaceuticals, Ferring Pharmaceuticals, Nestle Health Sciences, ProbioTech USA, Regeneron, Sanofi, and Takeda. D.M.B. has served as a speaker or adviser for AbbVie, Ardelyx, Ironwood Pharmaceuticals, and Salix Pharmaceuticals; has served as a consultant for Alnylam Pharmaceuticals, Bayer, Bioamerica, CinPhloro Pharma, Dr. Reddy's Laboratories, Gemelli Biotech, Laborie Medical Technologies, and Vibrant Gastro; has unvested stock options in Owlstone Medical and SideBy Care; and is on the Board of Directors for the International Foundation for Gastrointestinal Disorders (IFFGD).

## Supporting information


**Table S1:** Patient demographics and baseline characteristics (ITT population).
**Figure S1:** Childbearing potential across the age range 40–60 years.
**Figure S2:** Patient disposition.

## Data Availability

AbbVie and Ironwood Pharmaceuticals are committed to responsible data sharing regarding the clinical trials we sponsor. This includes access to anonymized, individual, and trial‐level data (analysis data sets), as well as other information (e.g., protocols, clinical study reports, or analysis plans), as long as the trials are not part of an ongoing or planned regulatory submission. This includes requests for clinical trial data for unlicensed products and indications. These clinical trial data can be requested by any qualified researchers who engage in rigorous, independent, scientific research, and will be provided following review and approval of a research proposal, Statistical Analysis Plan (SAP), and execution of a Data Sharing Agreement (DSA). Data requests can be submitted at any time after approval in the US and Europe and after acceptance of this manuscript for publication. The data will be accessible for 12 months, with possible extensions considered. For more information on the process or to submit a request, visit the following link: https://vivli.org/ourmember/abbvie/ then select “Home”.
