# Supplementary material for: Effect of Age and Age‐Defined Menopausal Status on Linaclotide Efficacy and Safety in Adults With Irritable Bowel Syndrome With Constipation or Chronic Idiopathic Constipation: Post Hoc Analyses of Phase 2b/3 Studies
Source: Neurogastroenterol Motil. 2026 Sep 6;38(9):e70429. doi: 10.1111/nmo.70429 (PMC13547636; doi:10.1111/nmo.70429)
Supplement: Supplementary file 1 [file NMO-38-e70429-s001.docx]

**Supplementary Information**

**TABLE S1** | Patient demographics and baseline characteristics (ITT population).

| **Parameter**^†^ | **Patients with IBS-C (*N* = 2348)** | | | | **Patients with CIC (*N* = 2400)** | | | | | |
| --- | --- | --- | --- | --- | --- | --- | --- | --- | --- | --- |
|  | **Aged < 65 years (*n* = 2185)** | | **Aged ≥ 65 years (*n* = 163)** | | **Aged < 65 years**  **(*n* = 148)** | | | **Aged ≥ 65 years**  **(*n* = 252)** | | |
|  | **PBO (*n* = 1087)** | **LIN 290 µg (*n* = 1098)** | **PBO (*n* = 84)** | **LIN 290 µg  (*n* = 79)** | **PBO (*n* = 885)** | **LIN 72 µg (*n* = 375)** | **LIN 145 µg (*n* = 888)** | **PBO (*n* = 110)** | **LIN 72 µg (*n* = 36)** | **LIN 145 µg (*n* = 106)** |
| Age, years | 42.7 (11.88) | 42.7 (11.85) | 70.4  (5.23) | 70.9  (5.10) | 43.8 (12.12) | 43.4 (12.31) | 44.7 (11.49) | 70.3  (4.88) | 71.4  (6.10) | 70.3  (4.60) |
| Median age, years (min, max) | 44 (18−64) | 43 (19−64) | 69 (65−87) | 70 (65−85) | 45 (18−64) | 44 (18−64) | 46 (18−64) | 69 (65−85) | 70 (65−90) | 69 (65−86) |
| Sex, female,  *n* (%) | 955 (87.9) | 973 (88.6) | 61 (72.6) | 56 (70.9) | 766 (86.6) | 290 (77.3) | 765 (86.1) | 84 (76.4) | 22 (61.1) | 73 (68.9) |
| Race, *n* (%) |  |  |  |  |  |  |  |  |  |  |
| White | 782 (71.9) | 798 (72.7) | 65 (77.4) | 63 (79.7) | 624 (70.5) | 267 (71.2) | 635 (71.5) | 98 (89.1) | 31 (86.1) | 88 (83.0) |
| Black or African American | 236 (21.7) | 227 (20.7) | 12 (14.3) | 8 (10.1) | 226 (25.5) | 88 (23.5) | 219 (24.7) | 10 (9.1) | 5 (13.9) | 16 (15.1) |
| Asian | 40 (3.7) | 49 (4.5) | 7 (8.3) | 7 (86.9) | 23 (2.6) | 14 (3.7) | 20 (2.3) | 1 (0.9) | 0 | 0 |
| Other | 29 (2.7) | 24 (2.2) | 0 | 1 (1.3) | 12 (1.4) | 6 (1.6) | 14 (1.6) | 1 (0.9) | 0 | 2 (1.9) |
| Ethnicity, n (%) |  |  |  |  |  |  |  |  |  |  |
| Hispanic or Latino | 174 (16.0) | 187 (17.0) | 11 (13.1) | 13 (16.5) | 224 (25.3) | 169 (45.1) | 220 (24.8) | 13 (11.8) | 9 (25.0) | 16 (15.1) |
| Not Hispanic or Latino | 913 (84.0) | 911 (83.0) | 73 (86.9) | 66 (83.5) | 661 (74.7) | 206 (54.9) | 668 (75.2) | 97 (88.2) | 27 (75.0) | 90 (84.9) |
| BMI, kg/m^2^ | 28.2 (6.36) | 28.6 (6.46) | 28.6 (6.86) | 27.7 (5.13) | 28.8 (6.53) | 29.0 (6.08) | 28.6 (6.09) | 28.2 (5.19) | 27.9 (5.76) | 28.6 (6.07) |
| CSBMs per week | 0.2 (0.49) | 0.2 (0.44) | 0.3 (0.53) | 0.4 (0.69) | 0.3 (0.51) | 0.2 (0.49) | 0.2 (0.45) | 0.3 (0.46) | 0.5 (0.71) | 0.5 (0.69) |
| Stool consistency^‡^ | 2.3 (0.98) | 2.3 (1.00) | 2.3 (0.92) | 2.5 (1.15) | 2.2 (1.04) | 1.9 (0.93) | 2.2 (1.00) | 2.3 (0.96) | 2.3 (0.88) | 2.5 (1.02) |
| Constipation severity^§^ | 3.8 (0.65) | 3.8 (0.66) | 3.7 (0.61) | 3.7 (0.67) | 3.6 (0.76) | 3.7 (0.77) | 3.5 (0.79) | 3.5 (0.68) | 3.6 (0.76) | 3.4 (0.77) |
| Straining severity^§^ | 3.5 (0.81) | 3.5 (0.81) | 3.3 (0.80) | 3.6 (0.78) | 3.4 (0.86) | 3.6 (0.86) | 3.4 (0.87) | 3.3 (0.89) | 3.5 (0.90) | 3.3 (0.76) |
| Abdominal pain score | 5.8 (1.73) | 5.8 (1.70) | 5.4 (1.84) | 5.5 (1.84) | 2.6 (1.07) | 2.8 (1.16) | 2.6 (1.09) | 2.5 (0.90) | 2.4 (1.09) | 2.5 (1.03) |
| Abdominal discomfort score | 6.2 (1.67) | 6.2 (1.63) | 5.8 (1.77) | 5.8 (1.83) | 2.9 (0.99) | 3.0 (1.07) | 2.9 (1.00) | 2.8(0.83) | 2.6 (1.03) | 2.7 (0.97) |
| Abdominal bloating score | 6.6 (1.83) | 6.7 (1.77) | 6.0 (1.85) | 6.0 (2.04) | 3.1 (0.94) | 3.2 (1.02) | 3.1 (0.96) | 3.0 (0.83) | 2.9 (0.98) | 2.9(1.00) |
| Overall abdominal symptom score | 6.2 (1.64) | 6.3 (1.59) | 5.7 (1.74) | 5.8 (1.80) | 2.9 (0.95) | 3.0 (1.04) | 2.9 (0.96) | 2.8 (0.80) | 2.6 (1.01) | 2.7 (0.96) |
| **Parameter**^†^ | **Age-defined premenopausal^¶^ (*n* = 801)** | | **Age-defined postmenopausal^¶^ (*n* = 807)** | | **Age-defined**  **premenopausal^¶^ (*n* = 700)** | | | **Age-defined**  **postmenopausal^¶^ (*n* = 954)** | | |
|  | **PBO (*n* = 399)** | **LIN 290 µg (*n* = 402)** | **PBO (*n* = 404)** | **LIN 290 µg (*n* = 403)** | **PBO (*n* = 298)** | **LIN 72 µg (*n* = 118)** | **LIN 145 µg (*n* = 284)** | **PBO (*n* = 389)** | **LIN 72 µg (*n* = 147)** | **LIN 145 µg (*n* = 418)** |
| Age, years | 35.4 (6.99) | 35.2 (7.23) | 56.0 (7.74) | 55.6 (7.31) | 35.3 (7.50) | 34.9 (8.01) | 36.1 (7.04) | 56.7 (7.97) | 56.8 (7.75) | 56.6 (7.68) |
| Median age, years (min, max) | 36 (18−45) | 36 (19−45) | 55 (46−87) | 55 (46−82) | 37 (18−45) | 37 (18−45) | 37 (19−45) | 55 (46−80) | 56 (46−82) | 55 (46−86) |
| Sex, female,  *n* (%) | 399 (100) | 402 (100) | 404 (100) | 403 (100) | 298 (100) | 118 (100) | 284 (100) | 389 (100) | 147 (100) | 418 (100) |
| Race, *n* (%) |  |  |  |  |  |  |  |  |  |  |
| White | 283 (70.9) | 291 (72.4) | 299 (74.0) | 311 (77.2) | 202 (67.8) | 86 (72.9) | 189 (66.5) | 296 (76.1) | 109 (74.1) | 316 (75.6) |
| Black or African American | 93 (23.3) | 93 (23.1) | 92 (22.8) | 77 (19.1) | 88 (29.5) | 29 (24.6) | 84 (29.6) | 82 (21.1) | 35 (23.8) | 92 (22.0) |
| Asian | 10 (2.5) | 10 (2.5) | 6 (1.5) | 8 (2.0) | nr | nr | nr | nr | nr | nr |
| Other | 13 (3.3) | 8 (2.0) | 7 (1.7) | 7 (1.7) | 8 (2.7) | 3 (2.5) | 11 (3.9) | 11 (2.8) | 3 (2.0) | 10 (2.4) |
| Ethnicity |  |  |  |  |  |  |  |  |  |  |
| Hispanic or Latino | 74 (18.5) | 79 (19.7) | 64 (15.8) | 57 (14.1) | nr | nr | nr | nr | nr | nr |
| Not Hispanic or Latino | 325 (81.5) | 323 (80.3) | 340 (84.2) | 346 (85.9) | nr | nr | nr | nr | nr | nr |
| BMI, kg/m^2^ | 28.1 (6.46) | 28.7 (6.75) | 29.1 (6.82) | 28.7 (6.31) | 29.2 (6.76) | 28.6 (6.28) | 28.7 (6.61) | 28.9 (6.20) | 30.2 (6.56) | 29.1 (6.17) |
| CSBMs per week | 0.2 (0.42) | 0.2 (0.42) | 0.2 (0.50) | 0.2 (0.47) | 0.2 (0.46) | 0.2 (0.47) | 0.2 (0.45) | 0.3 (0.53) | 0.3 (0.57) | 0.2 (0.50) |
| Stool consistency^‡^ | 2.2 (0.98) | 2.2 (0.99) | 2.4 (1.02) | 2.3 (1.05) | 2.2 (1.04) | 1.8 (0.92) | 2.2 (1.07) | 2.2 (1.04) | 2.0 (0.95) | 2.3 (1.01) |
| Constipation severity^§^ | 3.8 (0.63) | 3.8 (0.68) | 3.8(0.62) | 3.8 (0.66) | 3.6 (0.77) | 3.7 (0.82) | 3.5 (0.81) | 3.5 (0.73) | 3.7 (0.73) | 3.5 (0.80) |
| Straining severity^§^ | 3.5 (0.81) | 3.5 (0.84) | 3.5 (0.82) | 3.6 (0.80) | 3.4 (0.91) | 3.7 (0.90) | 3.4 (0.88) | 3.4 (0.84) | 3.6 (0.79) | 3.4 (0.84) |
| Abdominal pain score | 5.9 (1.74) | 5.9 (1.75) | 5.9 (1.77) | 5.8 (1.73) | 2.7 (1.06) | 2.9 (1.17) | 2.6 (1.11) | 2.5 (1.01) | 2.7 (1.13) | 2.5 (1.10) |
| Abdominal discomfort score | 6.3 (1.69) | 6.3 (1.66) | 6.3 (1.68) | 6.2 (1.69) | 2.9 (0.98) | 3.0 (1.10) | 2.9 (1.01) | 2.9 (0.95) | 2.9 (1.08) | 2.9 (1.02) |
| Abdominal bloating score | 6.7 (1.81) | 6.8 (1.76) | 6.7 (1.82) | 6.7 (1.80) | 3.2 (0.92) | 3.3 (1.01) | 3.2 (0.97) | 3.1 (0.92) | 3.1 (1.04) | 3.1 (0.98) |
| Overall abdominal symptom score | 6.3 (1.63) | 6.3 (1.61) | 6.3 (1.65) | 6.2 (1.62) | 2.9 (0.95) | 3.1 (1.05) | 2.9 (0.97) | 2.8 (0.90) | 2.9 (1.05) | 2.8 (0.98) |

Data are for the ITT population, defined as randomized patients who received at least one dose of study drug. For each parameter, values are presented for patients with available baseline data for that parameter.

^†^Data are mean (standard deviation) unless stated otherwise.

^‡^Measured using the Bristol Stool Form Scale.

^§^Constipation and straining severity were measured weekly and daily, respectively, using a 5-point ordinal scale (1 = not at all; 2 = mild straining; 3 = moderate straining; 4 = severe straining; 5 = extremely severe straining).

^¶^Female patients aged ≤ 45 years were age-defined premenopausal; female patients aged > 45 years were age-defined postmenopausal.

BMI, body mass index; CIC, chronic idiopathic constipation; CSBM, complete spontaneous bowel movement; IBS-C, irritable bowel syndrome with constipation; ITT, intent-to-treat; LIN, linaclotide; nr, not reported; PBO, placebo.

**FIGURE S1** | Childbearing potential across the age range 40–60 years.

Data on childbearing age from a phase 3 trial (NCT03573908) and a phase 2b trial (NCT02559206) of women with IBS-C treated with placebo or linaclotide. IBS‑C, irritable bowel syndrome with constipation.

**FIGURE S2** | Patient disposition.

^a^*n* is the number of randomized patients in each of the included studies in the *post hoc* analyses.

^b^Comprised female patients who were not receiving contraception or hormone replacement therapy during the intervention period; female patients aged ≤ 45 years were age-defined premenopausal; female patients aged > 45 years were age-defined postmenopausal.

CIC, chronic idiopathic constipation; IBS-C, irritable bowel syndrome with constipation; ITT, intent-to-treat; LIN, linaclotide; PBO, placebo.
